# Uncommon Urinary Actinomycosis Mimicking Upper Urinary Tract Urothelial Tumor: Case Report and Literature Review

**DOI:** 10.3390/microorganisms13051033

**Published:** 2025-04-30

**Authors:** Patricia A. Meza-Meneses, Rodrigo Pérez Becerra, Gerardo Garza Sainz, Luis Trujillo Ortiz, Adrián Martinez Correa, Alan Rodrigo Pérez Soriano, Ruben Miguel Angel Santiago González, Aarón Delgado Corral, Omar Vieyra Valdez, Genaro Argüelles Morales, Mario Alberto Toledo Díaz, Alberto Saldivar Luna, Carlos Alberto Castro-Fuentes, Victor Osornio Sánchez

**Affiliations:** 1 Infectology Service, Hospital Regional de Alta Especialidad de Ixtapaluca, Servicios de Salud del Instituto Mexicano de Seguro Social Para el Bienestar (IMSS-BIENESTAR), Carretera Federal Mexico-Puebla Km 34.5, Ixtapaluca 56530, Mexico; patricia_meza@ymail.com; 2 Urology Service, Hospital Regional de Alta Especialidad de Ixtapaluca, Servicios de Salud del Instituto Mexicano de Seguro Social Para el Bienestar (IMSS-BIENESTAR), Carretera Federal Mexico-Puebla Km 34.5, Ixtapaluca 56530, Mexico; becerra151@yahoo.com.mx (R.P.B.); gasg1975@hotmail.com (G.G.S.); drtrujillo68@gmail.com (L.T.O.); adriiano13@gmail.com (A.M.C.); alanarpsmd@gmail.com (A.R.P.S.); sagrm@outlook.com (R.M.A.S.G.); adcorral1911@gmail.com (A.D.C.); dr.vieyra@hotmail.com (O.V.V.); arguellesgenaro@gmail.com (G.A.M.); matoledodiaz@gmail.com (M.A.T.D.); saldivar.luna10@gmail.com (A.S.L.); 3 Research Unit, Hospital Regional de Alta Especialidad de Ixtapaluca, Servicios de Salud del Instituto Mexicano de Seguro Social Para el Bienestar (IMSS-BIENESTAR), Carretera Federal Mexico-Puebla Km 34.5, Ixtapaluca 56530, Mexico

**Keywords:** urinary actinomycosis, urothelial tumor, urinary tract, case report, misdiagnosed, mimicking

## Abstract

Urinary actinomycosis is a rare condition, often mimicking a urinary tract tumor. Due to its low prevalence, it can be challenging to diagnose and may be mistaken for malignancies. A 33-year-old female patient with a history of type 2 *Diabetes Mellitus* and recurrent urinary tract infections presented to the emergency room with right renal fossa pain radiating to the right hypochondrium, fever with chills, nausea, and vomiting. Physical examination revealed a positive Giordano sign and tenderness at the ipsilateral middle and upper ureteral points. A contrast-enhanced CT scan showed a mass infiltrating the distal third of the right ureter, causing retrograde dilatation and hydronephrosis. Additionally, a liver injury with both liquid and solid components was observed. Therefore, given the suspicion of a urothelial tumor, a diagnostic cystoscopy and ureteroscopy were performed. Using interventional radiology, an abscessed liver lesion was drained, yielding purulent fluid. The histopathological examination revealed no evidence of malignancy. However, due to the strong suspicion of upper urinary tract urothelial carcinoma, a right radical nephroureterectomy with bladder cuff excision and retroperitoneal lymphadenectomy was performed. Histopathological examination ultimately confirmed urinary actinomycosis. Consequently, antibiotic therapy with oral amoxicillin 2 g every 12 h was initiated, leading to a good clinical response. Despite its low incidence, urinary actinomycosis should be considered as a differential diagnosis in cases suspected of urothelial tumors in the upper urinary tract. Increased awareness of this rare condition may help prevent unnecessary surgical interventions.

## 1. Introduction

The genus *Actinomyces* belongs to the *Actinobacteria* phylum and *Actinomycetales* order and is comprised of anaerobic, Gram-positive, commensal bacteria found in the skin, oral cavity, gastrointestinal tract, and female genital tract [1]. To date, 25 species of Actinomyces have been described; of these, *A. israelii*, *A. gerencseriae*, *A. odontolyticus*, *A. meyeri*, *A. naelundii*, *A. viscosus*, *A. pyogenes*, *A. georgiae*, and *A. graevenitzii* are the main etiologic agents of actinomycosis in humans [1,2].

Actinomycosis is a rare chronic bacterial infection that occurs due to the migration of *Actinomyces* spp. to deep tissues through trauma, procedures, or foreign bodies that disrupt the barrier formed by the mucous membranes [2]. It commonly affects the cervicofacial area, thoracic, abdominal, and pelvic regions, and even the central nervous system (CNS). Among the unusual clinical forms of human actinomycosis, the involvement of the urinary tract is one of the least frequent. In this sense, *A. israelii*, *A. turicensis*, *A. naeslundii*, *A. ontolyticus*, and *A. gerencseriae* are the species most frequently identified in the genital tract, with few reports of *A. urogenitalis* and *A. neuii* in the amniotic fluid, as well as *A. meyeri* as a cause of chorioamniotitis [2]. Particularly, *A. schaalii*, *A. turicensis*, *A. urogenitalis*, *A. neuii*, and *A. europaeus* have been identified as etiological agents in urinary tract infections in humans [2].

Characteristic features of actinomycosis include chronic manifestations, abscess formation with sinus tracts, and purulent discharge. However, little is known about the virulence factors of *Actinomyces* species. However, they do not produce classical exotoxins, and their virulence is attributed to the ability to evade phagocytosis by the host immune system, to bind to collagen via fimbriae, and to develop biofilms [2,3].

At present, the prevalence of infections caused by *Actinomyces* species is unknown, and information in developing countries is incomplete [4] and often underdiagnosed due to their indolent nature and the difficulty in culturing the organism. In existing reports in the literature, the diagnosis of actinomycosis is based solely on clinical and histopathological findings; when present, sulfur granules can be stained with Gram stain or methylene blue and assessed by microscopy. However, granules are not specific for actinomycosis. So, on certain occasions, it can mimic other pathologies, like upper urinary tract urothelial tumor [5].

Therefore, this study aims to report a case of urinary tract actinomycosis that presented clinically as a urothelial tumor and resolved successfully. A literature review was also conducted to compile the clinical characteristics of reported cases of actinomycosis affecting the urinary tract.

## 2. Case Presentation

**Figure 1:**
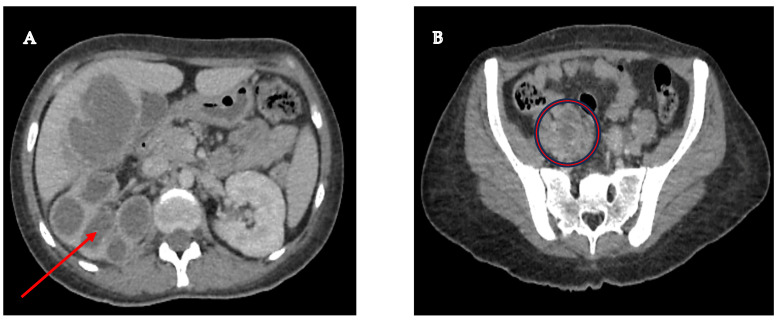
(**A**) Right kidney with significant cortical thinning, accompanied by ureterohydronephrosis (red arrow), suggesting a possible chronic obstruction of the urinary tract; (**B**) Pelvic mass measuring 46 × 25 mm, seemingly originating in the lumen of the right ureter, with infiltration into its distal third (red circle), a finding that raises a differential diagnosis between a urothelial tumor and a severe inflammatory infectious process.

A 33-year-old female patient had a history of type 2 *Diabetes Mellitus* under suboptimal control and without other risk factors for immunocompromise, with a history of recurrent urinary tract infections and use of multiple outpatient antibiotic regimens in the last year before admission. The patient arrived at the emergency room with a condition characterized by pain in the right renal fossa that radiated to the right hypochondrium, fever with chills, nausea, and vomiting. Upon arrival at our hospital, the vital signs showed blood pressure of 113/64 mmHg, temperature of 38.3 °C, heart rate of 99 bpm, respiratory rate of 21 rpm, and oxygen saturation of 97% in room air. Physical examination revealed a positive Giordano’s sign and positive ipsilateral middle and upper ureteral points. Therefore, laboratory studies were requested, where it was identified as iron deficiency anemia with hemoglobin of 10.3 g/dL, leukocytes of 10,500 cells/mcl with neutrophilia of 78%, platelets of 614,000/mcl, metabolic decontrol with fasting glucose of 240 mg/dL, preserved renal function with creatinine of 0.78 mg/dL, and elevated alkaline phosphatase of 156 IU/L and gamma-glutamyl transpeptidase of 51 IU/L. Additionally, the general urine examination showed negative nitrites, erythrocytes 10–25 by field, leukocytes 5–10 by field, epithelial cells 2–5 by field, and abundant mucoid filaments, suggesting urinary tract infection. Therefore, antibiotic coverage with ceftriaxone was started after collecting blood and urine samples. Additionally, as part of the care protocol, at least two peripheral blood culture vials (10 mL) were processed. Blood cultures were performed in BD Bactec™ Plus Aerobic/F and BD Bactec™ Anaerobic/F culture vials and incubated at 35 ± 2 °C in the BD BACTECT™ Blood Culture System (Becton Dickinson, Franklin Lakes, NJ, USA). However, the etiologic agent was not successfully isolated from the blood cultures. A urine sample was cultured in 5% sheep blood agar and MacConkey agar, yielding the same results as the blood cultures. A contrast-enhanced tomographic study highlighted a tumor that infiltrated the distal third of the right ureter with retrograde dilatation, an ipsilateral kidney with hydronephrosis and pyeloureteral dilatation, and a liver lesion with a liquid and solid component (Figure 1).

**Figure 2:**
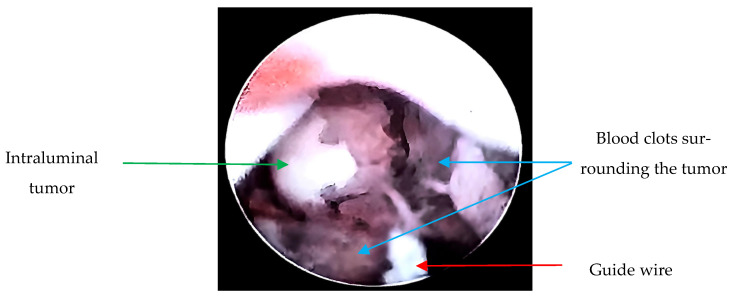
Ureteroscopy: Endoscopically, a guide wire (red arrow) and a tumor dependent on the lumen of the right ureter in the lower third with obstruction (green arrow) are observed. Additionally, blood clots surrounding the tumor are visualized (blue arrows). Upon passage of the guide wire, pyuria is noted due to the tumor causing complete obstruction, resulting in a foggy appearance.

Due to the suspicion of a urothelial tumor, the patient underwent diagnostic cystoscopy and ureteroscopy; during the procedure, a biopsy was taken (Figure 2). Using interventional radiology, it was decided to drain the abscessed liver tumor, obtaining purulent fluid. The drained fluid from the liver abscess was inoculated into liquid thioglycolate medium (Bioxon) and incubated for 24–48 h, then subcultured in BD Bactec™ Plus Aerobic/F and BD Bactec™ Anaerobic/F culture vials, replated, and incubated in a reducing environment; however, no isolation of *Actinomyces* spp. was obtained.

The patient completed 14 days of antibiotic treatment with ceftriaxone, and due to his positive progress with resolution of the systemic inflammatory response, the patient was discharged pending histopathological results.

**Figure 3:**
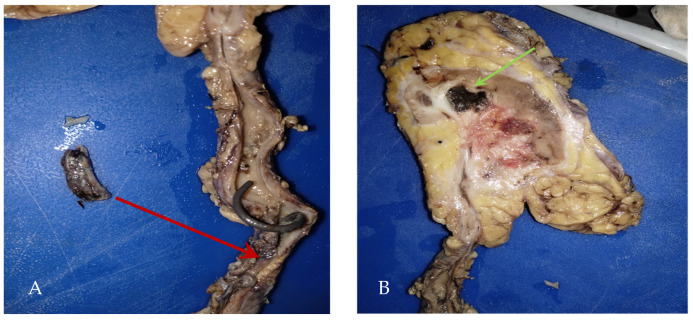
(**A**) Product of radical right nephroureterectomy with bladder cuff (290 g): calyces and renal pelvis with the presence of abundant inflammatory exudates and ureter tumor in the lower third (red arrow) with a report of intraluminal fibrosis without infiltrating the wall, (**B**) Macroscopic: renal abscess (green arrow).

During a follow-up visit, the histopathological examination revealed no evidence of malignancy, and given the high suspicion of urothelial cancer of the upper urinary tract, it was decided to subject the patient to a radical right nephroureterectomy with bladder cuff and retroperitoneal lymphadenectomy (Figure 3).

**Figure 4:**
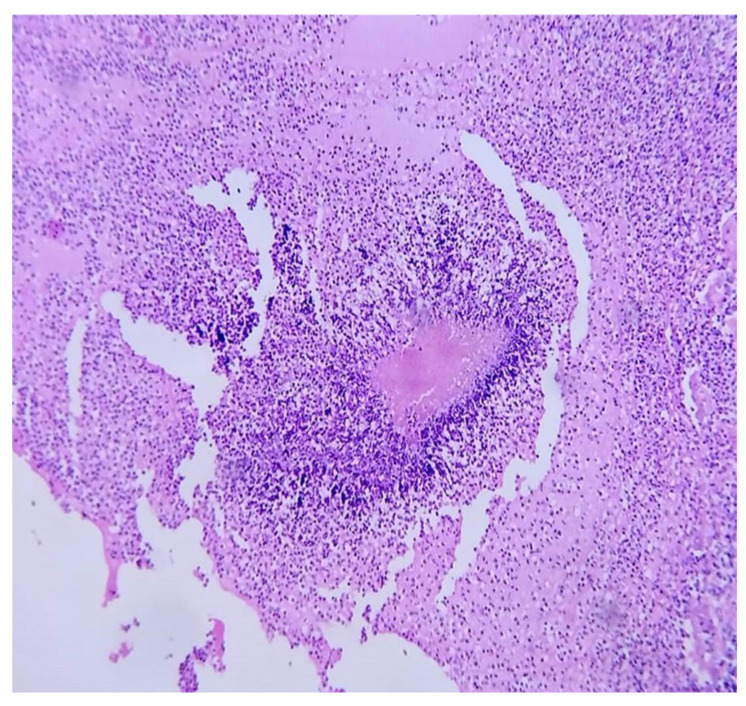
Microscopic renal abscess: hematoxylin-eosin staining at 40×, where leukocyte infiltration is observed in the interface with filamentous bacillary structures arranged in a radial and disordered manner and a deposit of eosinophilic material.

A few weeks later, the histopathological report of renal abscess showed filamentous bacillary structures arranged in a radial and disordered manner, deposit of eosinophilic material, and sulfide structures presumptive of *Actinomyces* spp. in hematoxylin-eosin staining (Figure 4), and due to the inconclusive diagnosis of malignancy, it was decided to adapt antibiotic therapy to amoxicillin at 2 g orally every 12 h for 6 months with good clinical evolution.

## 3. Discussion

In the present case report, we highlight a case of uncommon urinary actinomycosis, a misleading diagnosis of upper urinary tract urothelial tumor.

A search in the PubMed and Scopus databases was performed for case reports or reviews without distinction of languages over a period of 29 years (1995–2024), with the keywords “actinomycosis”, “*Actinomyces*”, “renal tumor”, “renal abscess”, and “*Xanthogranulomatous pyelonephritis*”. Table 1 compiles the 24 reported cases of actinomycosis with urinary tract involvement.

Of the total number of cases identified in the international literature, the main clinical presentation was a renal tumor; however, according to reports, it can also present as emphysematous pyelonephritis, xanthogranulomatous pyelonephritis, or even be asymptomatic. In addition to our clinical case, there is only one other report with a clinical presentation of a urothelial tumor with signs of obstruction and suspected neoplasia [13]. It is worth mentioning that clinical manifestations usually present in a subacute or chronic form. However, they have presented in a severe acute form, with the development of septic shock [19,24,26].

According to the literature, actinomycosis can occur in immunocompromised and immunocompetent patients. In the review of the reported cases, 44% (n = 11) presented some type of immunocompromise, such as *Diabetes Mellitus* (n = 7; 28%), pregnancy (n = 2; 8%), cancer (n = 1; 4%) and ESRD (n = 1; 4%), and Kidney stones were present in three cases (12%) as a risk factor for urinary tract infection.

Actinomycosis of the urinary tract has a good prognosis, with resolution of the infection. A total of 68% (n = 17) of patients underwent nephrectomy for the treatment of actinomycosis, and only 24% (n = 6) achieved resolution with antibiotic treatment or abscess drainage; only one patient died [26]. However, the patient suffered from various uncontrolled comorbidities and severe hyperkalemia that led to cardiac arrest.

The imaging findings of renal actinomycosis have been extrapolated from abdominopelvic actinomycosis, characterized by heterogeneous infiltrating solid and/or cystic masses that enhance with contrast medium. These findings are not different from renal masses from other etiologies, such as neoplastic [30,31]. The aggressive nature of this infiltration is the telltale clue to actinomycosis, as it is known for its ability to spread contiguously across tissue planes. In the cases found in the databases, 40% (n = 10) showed involvement of other tissues, including the duodenum, psoas muscle, liver, colon, lung, and diaphragm. Because *Actinomyces* species generally do not spread lymphatically due to the size of the organism, actinomycosis is rarely associated with regional lymphadenopathy. Thus, to distinguish actinomycosis from malignancy, the diagnosis of actinomycosis should be considered if a highly infiltrative contrast-enhancing mass is observed, accompanied by none or a few reactive regional lymph nodes [30]. In the cases reported in the literature, no involvement of the lymph nodes has been observed.

Currently, ureteroscopic biopsy is the most accurate method for the histological evaluation of oncological and non-oncological pathologies of the upper urinary tract. This technique can be used to diagnose urinary tract urothelial carcinoma (UTUC), and in some cases, staging can be established. However, in some cases, when the sample size is small, it is insufficient for an accurate diagnosis, or the sample is lost during processing [32].

Regarding the most commonly used ureteroscopy devices, these are baskets and forceps. In the diagnosis of urothelial carcinoma of the urinary tract, the use of a basket allows for successful cytopathological diagnosis in 94% of cases, compared to only 63% with forceps. For large tumors (≥10 mm), basket biopsy remains superior, providing a diagnosis in 94% of cases, compared to 80% with forceps [33].

In cases where ureteroscopic biopsy is inconclusive of malignancy, the origin of the disease should be suspected based on imaging studies and clinical course. Because there are no reports in the literature on the diagnostic accuracy of ureteroscopic biopsy specifically for the diagnosis of *Actinomyces* sp. [34].

The definitive diagnosis of actinomycosis requires a combination of histopathological, microbiological, and molecular studies.

In the cases reported to date, according to the primary data, the diagnosis was made by histopathological examination in 84% (n = 21). The presence of branching gram-positive bacilli should raise suspicion of actinomycosis; it is distinguished from other branching gram-positive bacilli, such as *Nocardia* spp., by its non-acid-fast nature [35]. Although in histopathological analysis, when present, sulfur granules can be stained with Gram stain or methylene blue and evaluated by microscopy; however, the granules are not specific for actinomycosis. Most reported cases of actinomycosis have been confirmed by histopathological examination, where the actinomycotic granuloma containing fibroblasts, plasma cells, giant cells, and polymorphonuclear cells is observed. In the present case report, the identification of the causative agent could only be performed at the genus level through the structures observed in the histopathological study, and because it was not possible to isolate the microorganism in a culture medium, identification at the species level was not possible.

Isolation of Actinomyces species in cultures was only reported in 20% (n = 5) of the cases found in the international literature, being caused by *A. israelii* (40%), *A. gerencseriae* (20%), *A. meyeri* (20%), and *A. odontolyticus* (20%). Hematogenous dissemination of actinomycosis has been reported to be rare in the reported cases, there was only one isolation of *Actinomyces* spp. in blood [22]. In the literature, it has been described that actinomycosis infections are polymicrobial in nature, but only two cases presented this characteristic with additional isolation of *Actinobacillus actinomycetemycomitans* [10] and *Proteus mirabilis* [29]. Perhaps the use of previous antibiotics for urinary tract infections is the cause of polymicrobial infections.

*Actinomyces* spp. are slow-growing microaerophilic bacteria, requiring prolonged anaerobic incubation of cultures for up to 20 days. However, the success rate in obtaining the etiological agent is low. Whereas, in cases where a positive culture is obtained, mass spectrometry such as matrix-assisted laser desorption/ionization-time of flight mass spectrometry (MALDI-TOF MS) can correctly identify up to 97% of isolates to the species level [3]. Alternative methods, such as 16S rRNA gene sequence analysis, have allowed the identification of *Actinomyces* spp. in clinical samples through the construction of phylogenetic relationships [36]. Furthermore, commercial kits with universal primers that amplify all members of the *Bacteria* domain are currently available [37].

Most *Actinomyces* species are susceptible to penicillin and other beta-lactams, and some are intrinsically resistant to metronidazole [38]. The literature reports that they may also be susceptible to cephalosporins, clindamycin, carbapenems, and tetracyclines; doxycycline [39] can be used as an alternative for patients allergic to penicillin. A total of 64% (n = 16) of cases reported in the literature were treated with some type of penicillin—benzylpenicillin, amoxicillin, or piperacillin.

Particularly, *A. europaeus* and *A. turicensis* are the species with the highest resistance to some antibiotics [39]. *A. turicensis* is resistant to clindamycin, tetracyclines (doxycycline and tetracycline), macrolides (clarithromycin and erythromycin), ciprofloxacin, and linezolid. Meanwhile, *A. europeaus* has shown resistance to ceftriaxone, clindamycin, macrolides (clarithromycin and erythromycin), ciprofloxacin, and tazobactam [39]. While an increase in the Minimum Inhibitory Concentration (MIC) values has been observed for *A. funkei* (tetracycline), *A. graevenitzii* (doxycycline and tetracycline), *A. israelii* (linezolid), *A. odontolyticus* (clindamycin), and *A. viscosus* (clindamycin) [40].

There are no treatment guidelines specifying the preferred duration or route of administration (oral or intravenous) for actinomycosis. Therefore, treatment should be individualized and usually prolonged, 6 to 12 months, until the infectious process is resolved. It is worth mentioning that abscess treatment requires drainage, while resective surgery may be performed in cases with extensive necrotic lesions or situations of antimicrobial therapy failure [2].

To our knowledge, this would be the 25th case report of actinomycosis with urinary tract involvement reported worldwide. Cases identified in the literature describe the potential for treating urinary actinomycosis with conservative management (antibiotics and abscess drainage) with good results [7,8,10,12,21,24], before considering more invasive interventions such as partial or radical nephrectomy. As in our case, the potential for misdiagnosis of urothelial cancer led to the decision to perform radical nephrectomy. Although good clinical outcomes are also reported for patients undergoing partial or radical nephrectomy in the reported cases, no case reported on the development of any potential long-term complications from this surgical procedure, such as the risk of developing chronic kidney disease, hypertension, or cardiovascular disease.

Among the limitations of this study are its retrospective nature, in which information on some cases is incomplete, and the description of imaging studies is inconsistent. By collecting only cases reported in the literature, these may not represent the entire population affected by urinary actinomycosis.

## 4. Conclusions

Due to the low growth success rate of *Actinomyces* spp. in culture media, histopathology represents a highly useful tool in the diagnosis of actinomycosis. Although the etiologic agent of *Actinomyces* was not identified at the species level in this case report, a favorable outcome was achieved with treatment with amoxicillin.

Actinomycosis can occur in patients with various risk factors, including DM, pregnancy, cancer, and ESRD. Kidney stones, on the other hand, are not the main predisposing factor for actinomycosis.

Differential diagnoses are necessary when urinary tract tumor masses are identified. Therefore, early and accurate diagnosis is crucial to avoid unnecessary surgical procedures and to initiate appropriate antimicrobial therapy.

## Figures and Tables

**Table 1:** Reported cases of actinomycosis with urinary tract involvement.

Gender	Age (Years)	Clinical Presentation	Imaging Findings	Diagnostic Method	Species	Risk Factors	Treatment	Outcome	Reference
Female	19	Renal tumor with duodenal fistula	US: Extensive tumor mass affecting the right kidney	Histopathology	Not identified	DM	Radical nephrectomy	Alive	[6]
Male	45	Renal tumor	US: 7-cm solid mass in the upper pole of the right kidney with less echogenicity than the renal cortex and with a multinodular internal pattern + poorly delimited subcutaneous lesion at the thoracic level CT: enlarged right kidney or with heterogeneous contrast enhancement and edema in the fascia, renal hilum, muscles, retroperitoneum and pleura	Histopathology	Not identified	None	IV penicillin for two months and then orally for at least 4 months	Alive	[7]
Male	41	Renal tumor	US: heterogeneous left renal mass that bulges around the contour and compromises the renal sinus and parenchyma. CT: Tissue mass infiltrating the left kidney with contrast enhancement + enlarged psoas muscle or with multiple ring-shaped enhancements + small focal hypodensity in the liver	Histopathology	Not identified	DM	Abscess drainage and a high dose of oral penicillin	Alive	[8]
Male	52	Renal tumor	US: left-sided renal mass CT: 4 cm mass in the upper pole region of the left kidney with an irregularly shaped radiolucent middle area	Histopathology	Not identified	None	Nephrectomy and oral amoxicillin for 3 months	Alive	[9]
Male	16	Renal tumor	CT: renal mass in the upper right pole + mass in the left lobe of the liver + multiple pulmonary nodules	Histopathology + Positive abscess culture	*Actinomyces israelii* + *Actinobacillus actinomycetemycomitans*	None	IV penicillin for two weeks, followed by oral doxycycline for 6 months	Alive	[10]
Male	68	Renal tumor	US: Hypoechoic solid mass of the right kidney CT: Enlarged right kidney with a mass with little enhancement with contrast medium MRI: Renal tumor of hypo to isointense (T1) and hypointense (T2)	Histopathology	Not identified	Oral infections	Nephrectomy	Alive	[11]
Male	64	Renal tumor	CT: heterogeneous mass with enhancement in the right flank containing air-fluid levels and an inflammatory reaction affecting the right colon and psoas muscle	Histopathology	Not identified		IV penicillin for 8 weeks followed by doxycycline + oral ciprofloxacin for 4 months	Alive	[12]
Male	66	Right ureter tumor	US: grade II ureteric opyelo-calicectasis of the right kidney CT: Hydronephrotic atrophy of the right kidney, with no intraureteral endoluminal images visible	Histopathology	Not identified	DM	Right radical nephroureterectomy	Alive	[13]
Male	41	Renal tumor	CT: large mass compatible with a large locally advanced left hypernephroma, with invasion of the left psoas and descending colon	Histopathology	Not identified	None	Nephrectomy	Alive	[14]
Female	25	Renal tumor	CT: Right renal solid mass that enhances with contrast with central liquefaction areas and invades the liver, alveolar infiltrates	Histopathology	Not identified	Pregnancy	Nephrectomy and oral penicillin for 2 months	Alive	[15]
Female	27	Renal tumor with retroperitoneal bleeding	US and CT: Right renal mass with retroperitoneal bleeding	Histopathology	Not identified	None	Nephrectomy	Alive	[16]
Male	39	Chronic kidney infection	CT: Enlargement of the left kidney due to an infiltrative process with enhancement + wall of the descending colon with increased thickness and enhancement + sinus tracts extending from the colon to the left iliopsoas muscle and the muscles of the abdominal wall	Histopathology	Not identified	None	Nephrectomy + segmental resection of the descending colon Oral penicillin for 6 months	Alive	[17]
Female	50	Kidney cyst	US: 8 × 8 cm left renal mass with a central fluid portion and a thick surrounding wall CT: Enlarged left kidney with a well-delineated 5 × 9 cm cystic lesion of homogeneous content of 0–3 HU surrounded by a thickened outer wall without contrast enhancement.	Positive culture of purulent discharge	*Actinomyces* *israelii*	None	Nephrectomy and oral amoxicillin for 3 months	Alive	[18]
Female	42	Septic shock caused by Emphysematous pyelonephritis	CT: striation of perirenal fat, calculus at the right ureteropelvic junction with formation of air and subcapsular fluid in the right kidney, mild right pleural effusion	Histopathology	Not identified	DM Kidney stones	Nephrectomy and oral penicillin	Alive	[19]
Male	55	Renal tumor	US: Heterogeneous mass in the upper pole of the left kidney CT: Mass in the upper pole of the left kidney with thickening of the renal fascia MRI: Hypointense mass on T1 and T2	Histopathology	Not identified	None	Nephrectomy and oral penicillin for 8 weeks	Alive	[20]
Female	80	Renal tumor	MRI: central hyperintense lesion with a surrounding isointense mass on T2-weighted images, suggestive of central necrotizing tumor CT: exophytic renal mass with extension contiguous to the diaphragm, pleural space and lower right lobe	Positive culture of purulent fluid	*Actinomyces* *gerencseriae*	Renal carcinoma	Oral penicillin G for 3 weeks and then amoxicillin for 6 months.	Alive	[21]
Female	59	Pyonephrosis	US: cystic renal mass with an irregular solid border CT: enlarged right kidney infiltrated with multiple cystic lesions with hypodense content	Positive blood culture + Histopathology	Not identified	DM ESRD	Nephrectomy and Penicillin for 4 weeks	Alive	[22]
Male	56	Asymptomatic	US: multiple stones in both kidneys	Histopathology	Not identified	Nephrolithiasis with ureteral catheterization	Right nephrectomy and doxycycline for 4 weeks	Alive	[23]
Female	75	Septic shock caused by Emphysematous pyelonephritis	CT: pyonephrosis of the right kidney, pneumaturia in the collecting system, cyst in the lower pole, dilated pelvicalyceal cavities CT: persistent posterior abscess of the right kidney with infiltration of the perinephric space	Positive urine culture	*Actimomyces meyeri*	None	Open stenting of the right ureter with drainage of Pyuria + percutaneous drainage of the abscess Amoxicillin for 3 months	Alive	[24]
Male	52	Septic shock with bilateral necrotizing papillitis	US: loss of corticomedullary differentiation and increased renal echogenicity. CT: enlarged kidneys with reduced nephrogenic density and compact pyelocaliceal system.	Histopathology	Not identified	DM	Cefotaxime and azithromycin, empirically	Deceased	[25]
Female	20	Renal tumor	US: Hemorrhagic mass in the right renal subcapsular area extending to the right diaphragm MRI: Heterogeneous mass in the right kidney extending to the edge of the liver and mild hydronephrosis, splenomegaly	Histopathology	Not identified	Pregnancy	Resection of retroperitoneal mass + partial nephretomy IV piperacillin /Tazobactam and subsequently amoxicillin/clavulanate for 6 months	Alive	[26]
Male	63	*Xanthogranulomatous pyelonephritis*	CT: Diffuse *Xanthogranulomatous pyelonephritis* in the left kidney with extension of inflammation to the pararenal tissues	Histopathology	Not identified	None	Not available	Not available	[27]
Male	8	Renal tumor + *Xanthogranulomatous pyelonephritis*	US: solid cystic mass with extension to perinephric adipose tissue CT: enlargement of the right kidney with thickening of Gerota’s fascia and heterogeneous mass with cystic component in the upper pole of the right kidney	Histopathology	Not identified	None	Right nephrectomy and amoxicillin for 4 months	Alive	[28]
Male	36	*Xanthogranulomatous pyelonephritis*	CT: left *Xanthogranulomatous pyelonephritis* with a staghorn calculus and a urocutaneous fistula	Histopathology and + Positive culture of purulent discharge	*Actinomyces odontolyticus Proteus mirabilis*	Kidney stones	Radical left nephrectomy IV ertapenem for 6 weeks	Alive	[29]
Female	33	Right ureter tumor	CT: Right kidney with decreased cortical thickness associated with ureterohydronephrosis + Pelvic tumor with apparent origin in the lumen of the right ureter with infiltration into the distal third + liver lesion with liquid and solid components	Histopathology	Not identified	DM	Nephrectomy and Amoxicillin for 6 months	Alive	**

US = ultrasound; CT = computed tomography; MRI = magnetic resonance imaging; DM = *Diabetes Mellitus*; ESRD = end-stage renal disease; IV = intravenous.

## Data Availability

The original contributions presented in this study are included in the article. Further inquiries can be directed to the corresponding authors.
